# A location privacy protection method based on blockchain and threshold cryptography

**DOI:** 10.1371/journal.pone.0324551

**Published:** 2025-06-04

**Authors:** Zhaowei Hu, Ruifang Jin, HangYi Quan, ShiYun Ni, Peng He

**Affiliations:** 1 School of Computer Science and Artificial Intelligence, Changzhou University, Changzhou, China; 2 College of Computer, Jilin Normal University, Siping, Jilin, China; 3 Zhou Youguang School of Chinese Language and Literature, Changzhou University, Changzhou, China; Jaramogi Oginga Odinga University of Science and Technology, KENYA

## Abstract

To address privacy leakage risks arising from low collaborative user engagement, third-party trust deficits, and insufficient collaboration timeliness in location-based services (LBS), this paper proposes a dual-protection framework integrating blockchain technology and threshold cryptography for safeguarding location privacy. The framework employs asymmetric encryption with Shamir’s (*t*, *n*) secret sharing to encrypt user queries, distributing decryption key fragments to collaborative users while generating *n* anonymous service requests through location generalization strategies. A temporary private blockchain constructed using smart contracts ensures confidential data transmission, supported by a dynamic privacy parameter configuration system based on Byzantine fault tolerance. The framework implements a priority-response consensus mechanism through Token-based equity proof-of-stake, prioritizing service for users with higher Token values. To mitigate privacy breaches caused by unresponsive collaborators, a competitive incentive mechanism ensures timely information submission. Through ciphertext fragment verification algorithms and Lagrange interpolation-based key reconstruction, the framework enables secure query decryption and service matching in untrusted third-party environments, guaranteeing information security, integrity, and non-repudiation. Experimental validation using real-world datasets confirms the framework’s feasibility and operational effectiveness.

## 1. Introduction

With the rapid development of location-based services (LBS), location privacy protection has become a major research focus. Existing methods such as k-anonymity and differential privacy protection predominantly rely on third-party intermediary servers for anonymization or noise injection [1–4]. However, these servers face reliability challenges due to performance bottlenecks and insecure data transmission protocols, creating potential privacy leakage risks. To address service bottlenecks and centralized attack vulnerabilities, scholars have proposed distributed architecture-based privacy protection approaches. In these systems, service requesters collaborate with neighboring users to generate collective anonymous areas or jointly submit queries, thereby safeguarding both location and query privacy. While these methods have expanded the theoretical framework of privacy preservation, they still exhibit security limitations in real-world implementations.

On the one hand, current location-based privacy protection systems exhibit fundamental limitations in their trust model and protection mechanisms. Existing frameworks predominantly adopt a dichotomous trust assumption: while treating location service providers as untrusted entities, they naively presume unconditional honesty among collaborating users in anonymous sets. However, the participants in collaborative anonymity schemes cannot be considered fully trustworthy, as they may intentionally or inadvertently disclose requesters’ sensitive geospatial data. This flawed trust model leads to critical vulnerabilities when requesters directly publish actual coordinates within anonymous regions. The prevailing protection paradigm adopts a segregated approach, either safeguarding location coordinates through spatial obfuscation or protecting query semantics via cryptographic encapsulation, but fails to implement concurrent protection for both privacy dimensions. This dichotomy creates multiple privacy leakage vectors. On the other hand, the existing k-anonymity model disregards the dynamic nature of collaborative participation. Practical implementations face operational challenges when insufficient participants satisfy the anonymity threshold (k-value), or when collaborators fail to submit required responses within temporal validity windows. Such scenarios induce anonymity set collapse, resulting in direct exposure of users’ private location-query associations.

This paper proposes a blockchain-enhanced and threshold cryptography for location privacy preservation to address three critical challenges in location-based services: untrusted third-party providers, unresponsive collaborators and delayed information propagation. The framework achieves provably secure protection for both spatial and query privacy through cryptographic method design. The main contributions of the study can be summarized as follows:

Firstly, to address the risks of geospatial data exposure stemming from semi-trusted collaborators and untrusted anonymous location service providers in location-based services, a threshold cryptosystem integrating Shamir’s (*n*,*t*) secret sharing is proposed. The framework encodes user queries into ciphertexts Q using requester’s private key s_k_. Through polynomial-based key splitting, the decryption key p_k_ is distributed as (k_i_,F(k_i_)) (i = 1,2,…,n) fragments to *n-1* collaborative nodes. The requester and collaborators send their real position, request ciphertext and key fragment to the LBS server respectively. Spatial obfuscation is achieved when ≥*t* fragments combine to reconstruct decryption key p_k_ for Q decryption. This dual-phase encryption satisfies IND-CCA2 security, preventing both location exposure (ε,δ)-differential privacy guarantee and query pattern inference.

Secondly, to solve the challenges of reluctant participation and delayed response in anonymous collaboration systems, a blockchain-based incentive mechanism for distributed privacy preservation is proposed. The framework employs blockchain smart contracts to establish permissioned blockchain networks, constructing cryptographically authenticated collaborator sets to ensure end-to-end confidentiality of data transmissions. Subsequently, a Proof-of-Entitlement mechanism is implemented, which uses Token value as the basis for whether user’s request can be responded preferentially, and the requester with the higher Token value is responded preferentially. The Token incentive mechanism is introduced, the first *t* cooperative users who send collaborative information will get a certain Token value. The framework uses competition mechanism to encourage cooperative users to send collaborative information in time, the time-bound reward system enforces strict response deadlines, effectively mitigating information leakage risks during anonymous collaboration through cryptographically enforced behavioral constraints.

Thirdly, to guarantee ciphertext integrity and unforgeability, we construct a verifiable secret-sharing scheme based on Shamir’s (*t*,*n*) threshold cryptography is constructed. To ensure information integrity and non-repudiation, the framework utilizes key partitioning technology to generate a verification key. By employing a cryptographic-based key verification algorithm combined with a secret sharing mechanism, it effectively validates the authenticity of ciphertext fragments. Through the experimental analysis on the real data set, the validity of the proposed approach is verified.

The structure of the paper is organized as follows: Section 2 reviews related work in the field. Section 3 details the proposed research model and its foundational concepts. Section 4 systematically presents the methodology and research design. and the effect of privacy protection is analyzed in section 5. The experiment and result analysis are shown in section 6, and section 7 summarizes the research contents and proposes potential directions for future research.

## 2. Related work

To safeguard users’ location privacy, researchers have developed many privacy-preserving methodologies [5–8], which h are divided into centralized protection mechanisms, distributed collaborative frameworks, and management mechanisms governing collaborative participation.

### 2.1. Centralized privacy protection method

The centralized privacy protection method was first proposed by Gruteser [9], where a central server is responsible for constructing anonymous areas, forming anonymous sets or adding disturbing noise before sending anonymized data to the LBS server for obtaining location-based services [10]. The most common methods include k-anonymity [11–13], dummy location [14,15], and differential privacy [16,17], with many improved location privacy protection methods being developed based on these approaches. Xu proposed a virtual location-based privacy protection method [18] that uses entropy to quantify anonymity and constructs a virtual location selection algorithm through effective distance-based location distribution feature extraction, which enhances the validity of virtual locations while improving the accuracy of interest point retrieval services. To protect location information and improve data availability in published trajectories, Jing developed a deep learning-based differential privacy protection method [19]. This approach employs a temporal graph convolutional network model to extract spatiotemporal features for budget matrix prediction, establishes privacy budget allocation rules based on recursive depth, and applies Laplacian noise to generate large-scale spatiotemporal trajectory data for protecting users’ location privacy. Addressing vulnerabilities to background knowledge attacks and edge information attacks in existing methods, Zhang proposed a service-based dummy location perturbation privacy protection method [20]. By using WordNet structure to ensure location semantic differences, discretizing and filtering dummy locations with Heron’s formula, and constructing secure anonymous sets according to anonymity levels, this method enhances location privacy security.

The centralized privacy protection method primarily relies on a central server to anonymize users’ location information. With strong data-processing capabilities, the central server compensates for mobile device’s limited computational capacity, thereby ensuring effective privacy protection. To address the adverse effects of location preferences and location semantics on privacy protection, Zhang proposed a differential privacy method based on location semantics and trajectory prediction [21]. This approach converts trajectory data into a prefix tree structure, classifies locations by calculating their sensitivity, allocates privacy budgets, and adds calibrated noise according to predefined protection levels, achieving a balance between privacy budgets and service quality. Regarding the challenges caused by discrete location data in privacy protection, Wang introduced a differential privacy-based method [22]. It employs a trajectory graph model and weighted frequent subgraph mining techniques to transform location data mining into frequent trajectory graph mining. By applying the Laplace mechanism to inject noise during subgraph mining and using the exponential mechanism to select anonymized datasets, this method enhances data utility while preserving privacy. Li developed a personalized location privacy protection method using location semantics [23]. It generates sensitivity-weighted documents based on semantic analysis, identifies optimal regions for k-anonymity through reinforcement learning, and implements bidirectional k-anonymity between user locations and query locations to safeguard privacy.

However, with the increase in mobile users, the amount of data processed by location-based services grows exponentially, making the central server both a performance bottleneck and a prime target for attacks in centralized privacy protection systems. On one hand, the process of all users submitting anonymous requests to the central server imposes substantial computation and communication overhead, establishing the server as a system performance bottleneck that degrades service quality. On the other hand, the central server’s storage of users’ location-sensitive information creates significant risks, if compromised by attackers, it could lead to massive privacy disclosure. Consequently, researchers now regard central servers as untrusted third parties, proposing either cryptography-based privacy protection methods [24–26] or distributed approaches leveraging decentralized architectures [27–29].

### 2.2. Distributed privacy protection method

In a distributed privacy protection architecture, requesting users can autonomously generate anonymous zones or collections to protect their location privacy by collaborating with other users. Huang proposed a method for requesting users to build anonymous areas with the help of collaborative users in Social Networks [28]. Sun classified users’ real locations and proposed a distributed privacy protection method based on location tags [29]. Ghinita used Hilbert curves to construct anonymous regions with collaborative users [30], when the number of collaborative users was large, the method employed B+ trees to find neighboring users for anonymous region construction [31]. Chow proposed a method to construct anonymous areas by using the real locations of historical collaboration users [32]. Peng argued that requesting users to send dummy queries simultaneously with service requests could obfuscate real queries to protect personal privacy [33]. To solve the privacy leakage and performance bottleneck problems of central servers, Zhang proposed a location trajectory privacy protection method with multi-anonymous-server threshold encryption [34]. This approach deploys multiple anonymizers between the user and the LBS location server, divides query content into n sub-information fragments sent to *n* randomly selected anonymizers, selects one anonymizer to perform k-anonymity on the user location, and forwards the query request to the LBS server. This method achieves distributed k-anonymity privacy protection for user query content while avoiding single point failure risks. However, since this approach only sends one real location and one authentic query content to the LBS server, the user’s private information remains at risk of disclosure.

The distributed structure of the blockchain provides a new way to solve the problem of user privacy protection. Liu pointed out that there may be location-sensitive information leakage and location deception among users participating in anonymous collaboration, he proposed a distributed k-anonymous location privacy protection method based on blockchain [35]. The method records location information provided by collaborative users through blockchain technology. If collaborative users are found to have location leakage and cheating behaviors, they cannot successfully construct anonymous areas as requesters, thereby restricting their selfish behaviors. To control access to transaction content in blockchain and achieve controllable privacy protection, Li proposed a blockchain-based controllable privacy protection method using attribute-based encryption [36]. This approach allows transaction organizations to customize personalized privacy protection policies and employs attribute-based encryption to control access to privacy protection traps, ensuring data legitimacy and security. Zhang proposed a privacy protection method based on information partitioning and blockchain smart contracts [37], which encrypts query information and uses a (*t*, *n*) threshold scheme for partitioning to prevent collusion attacks. Yang developed a blockchain-based privacy protection method for user location semantics [38], automatically collecting location semantic information through smart contracts to enhance the security of anonymous data collection. Zhu introduced a blockchain-based location sharing privacy protection method [39] that uses Merkle Tree to divide location regions, converts precise locations into regional approximations, and shares these regions with service requesting users to achieve location privacy protection.

However, there are some security problems in privacy protection methods with collaborative user participation in a distributed environment. Firstly, collaborative users are not completely trusted, they may reveal user privacy information, or attackers may masquerade as collaborative users to obtain the requesting users’ private data. Secondly, other users around the requesting user are not necessarily willing to participate in anonymous collaboration, nor can they always complete anonymous information processing in time. These factors may lead to the failure of anonymous methods and subsequent disclosure of private information. Therefore, requesting users directly publishing real location information and sharing requested content with collaborative users creates risks of privacy leakage. The willingness of users to collaborate and the timeliness of their collaborative processing remain key factors in protecting requesting users’ privacy from disclosure.

### 2.3. Management mechanism of collaborative user participation

In current location privacy protection methods for distributed environments, it is assumed that cooperative users are honest and trustworthy and willing to participate in anonymous collaboration with requesting users. However, in practical applications, malicious cooperative users may disclose sensitive information such as the requesting user’s location and query content, leading to privacy breaches. To address these issues, researchers have proposed privacy protection mechanisms based on cooperative user management and behavioral constraints. Li proposed a reputation-based incentive method for privacy protection [27], which introduces reputation metrics into anonymous zone construction. Cooperative users can improve their reputation values by helping requesting users build anonymous zones, thereby encouraging honest participation. Liu developed a constraint mechanism for collaborative user participation [35] that penalizes location disclosure and fraudulent behavior, preventing self-interested users from successfully anonymizing as requesters. Zhang implemented a competitive incentive mechanism using smart contracts [37], enabling collaborative users to actively submit cooperative information alongside initial users. Yang introduced a privacy protection method for single-round sealed double auctions [40], which motivates users to participate in anonymous collaboration and construct anonymous regions.

To sum up, in current location-based privacy protection approaches, the user’s location information is protected so that other participants cannot access the specific location data, while still allowing them to obtain the query content. Alternatively, the user’s query information is protected to prevent others from knowing the query’s specific content, though they may still discern the user’s precise location. These approaches preserve privacy by severing the link between location and query information, yet they fail to provide simultaneous protection for both location privacy and query privacy. Current methods also face privacy leakage risks stemming from untrustworthy location services, uncooperative users, and delayed information transmission. To address these issues, this paper proposes a blockchain and threshold cryptography-based location privacy protection method. By employing a secret sharing algorithm, threshold encryption system, token incentive mechanism, blockchain consensus protocol, and smart contracts, the proposed approach effectively mitigates threats including insufficient trustworthiness, collusion attacks among collaborative users, weak cooperation incentives, and delayed anonymity provision in existing systems, thus achieving dual protection of both location and query privacy.

The main differences between the work done in the paper and the current research are as follows: Firstly, by using threshold cryptography, a location privacy protection mechanism based on secret sharing is constructed. It employs the Shamir (*t*, *n*) secret sharing method to divide the key and the Lagrange interpolation algorithm to recover the key. This mechanism encrypts query content, splits decryption keys, and generalizes real locations, addressing the issue of incomplete trust in third parties within location-based services while achieving dual protection of location and query privacy. Secondly, a distributed privacy protection mechanism based on blockchain is developed through blockchain theory and methods. It utilizes smart contracts to create temporary collaborative private chains, ensuring confidentiality in information transmission. Token value serves as proof of rights to establish a user consensus mechanism that prioritizes requests with higher Token values. Additionally, a Token incentive mechanism with a competition framework encourages collaborative users to promptly submit information, resolving privacy leakage caused by anonymous collaborators’ reluctance to participate or delayed responses. Thirdly, verification keys are integrated into the processes of information encryption, key partitioning, data transmission, and key reconstruction. Key verification algorithms and share union algorithms are applied to authenticate information integrity and validity. The Byzantine fault-tolerant mechanism enhances robustness by setting privacy protection parameters. A public permission chain is established through blockchain admission mechanisms, with temporary private chains subsequently created on this foundation. This structure further narrows the scope of potential user privacy exposure and mitigates disclosure risks caused by collusion attacks.

## 3. Model foundation and basic method

### 3.1. Model foundation

#### 3.1.1. Secret sharing method.

Secret sharing is a method of securely distributing secrets among multiple participants by splitting a secret S into multiple shares. Each participant independently holds one share, and only when a specific threshold number of shares are combined can the secret S be reconstructed. If the obtained shares are fewer than this threshold, no information about the secret S can be revealed regardless of the combination strategy employed.

Shamir’s (t,n) secret sharing scheme, implemented by constructing a polynomial of degree *t*-1, divides the secret S into *n* distinct shares distributed to n participants. The secret can only be recovered when at least *t* shares are available. The specific process involves:

① **initialization**. set *n*, *t* as positive integers, and t ≤ n, secret distributor D randomly selects *n* different non-zero elements x_1_,x_2_,…,x_n_ from finite field GF(p) to identify *n* participants U_r_={U_1_,U_2_,…,U_n_} (r = 1,2,…,n), and discloses x_r_ and corresponding U_r_.
② **Secret distribution.** The secret S ∈ Z_q_ (q is a large prime) is decomposed into *n* pieces {s_1_,s_2_,…,s_n_}, and then these pieces s_i_(1 ≤ i ≤ n) are distributed to *n* participants U_r_={U_1_,U_2_,…,U_n_}. Any (t-1) elements a_i_(i = 1,2,…,t-1) in GF(p) are chosen to construct a polynomial of degree *t*-*1*, and each fragment is a coordinate point (x_i_,F(x_i_)) of the polynomial, which generates the polynomial:

$$F(x)=\sum_{i=1}^{t-1}a_{0}+a_{i}x^{i}modp$$ (1)

where p is a large prime and p > s, secret s = F(0)=a_0_.

Generate n sub-secrets for all U_r_ ∈ U:


$$s_{r}=F\left(x_{r}\right)=\sum_{i=1}^{t-1}a_{0}+a_{i}x^{i}modp(r=1,2,\ldots ,n)$$ (2)

Then s_r_ is securely sent to the appropriate U_r_.

③ **Secret recovery.** Any *t* fragment holders {U1,U2,…,Ut} combine their fragments and use the Lagrangian polynomial interpolation algorithm to recover the secret S:

$$\begin{array}{c} S=F(0)=\sum_{i=1}^{t}f\left(x_{i}\right)\prod_{v=1,v\neq l}^{t}\frac{-x_{v}}{x_{l}-x_{v}}modp \end{array}$$ (3)

***Example***. When n = 7, t = 5, construct a polynomial of order t-1 = 5−1 = 4, i.e.,


$$F(x)=a_{0}+a_{1}x+a_{2}x^{2}+a_{3}x^{3}+a_{4}x^{4}$$

**Fig 1:**
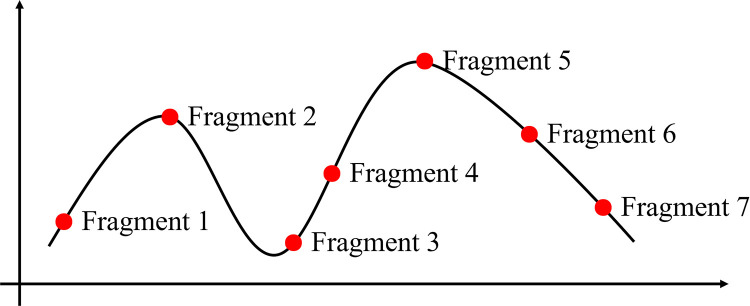
Secret sharing example.

The secret represented by the curve is divided into 7 pieces, as shown in Fig 1. When any 5 pieces of the 7 pieces are obtained, the coefficients of the polynomial F(x) can be inversely derived, and the constant coefficient a_0_ can be determined, the secret value can be obtained.

#### 3.1.2. Threshold encryption scheme.

**Fig 2:**
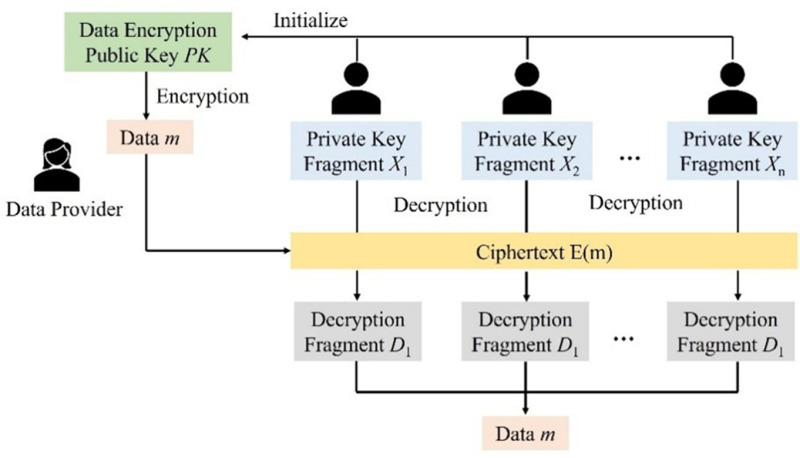
Threshold encryption flow.

A threshold encryption scheme allows any user to encrypt information using the public encryption key, while the private decryption key is split into multiple shares distributed among key holders. Only when a threshold number of key holders collaborate to combine their shares can the decryption key be reconstructed, enabling the encrypted information to be decrypted. The specific process is illustrated in Fig 2.

① **Initialize**. The secret owner obtains the key pair (PK, SK), PK is the public key, SK is the private key, and sets the number *n* of private keys to be divided and the minimum parameter *t* of key recovery. Then the private key SK is divided into *n* key fragments sk_i_ and distributed to *n* participants.
② **Encryption**. The data owner encrypts the plaintext *m* using the public key PK to generate the ciphertext E(m).
③ **Generating decrypted fragments**. Participants decrypt the ciphertext E(m) using their own private key fragments sk_i_ to generate decrypted fragments D_i_(m).
④ **Aggregation and decryption of fragments**. The participant aggregates the decrypted fragments D_i_(m), and when the obtained decrypted fragments D_i_(m) are not less than *t*, the aggregation and decryption can be completed to obtain the data plaintext *m*.

### 3.2. Basic method

This paper addresses security threats in existing privacy protection systems including trust deficiency, collusive attacks among cooperative users, weak collaboration incentives, and delayed anonymization by proposing a blockchain-based location privacy protection method with threshold cryptographic mechanisms. The solution mitigates privacy leakage risks stemming from uncooperative participants, unreliable location services, and delayed information exchange, achieving dual protection for both location and query privacy. The method employs Shamir’s (t, n) secret sharing and threshold encryption to secure requesters’ query content. After splitting the decryption key into *n* shares, the key shares and encrypted requests are distributed to *n* − 1 cooperative users. All *n* participants then collectively forward these components to the LBS server. Through location/query obfuscation enabled by *n* − 1 cooperating users, neither semi-trusted collaborators nor the LBS server can access the requester’s private data. Each collaborator only transmits encrypted queries and a single key share, ensuring no entity obtains complete user information.

The LBS server can reconstruct the decryption key through the Lagrange interpolation algorithm, enabling decryption of the request ciphertext to access the user’s actual query content. It also obtains location data from both the requesting user and *n*-1 cooperative users. However, the server cannot determine which specific location corresponds to the genuine user, nor which particular user initiated the query request. Consequently, the real user’s private information remains protected.

**Fig 3:**
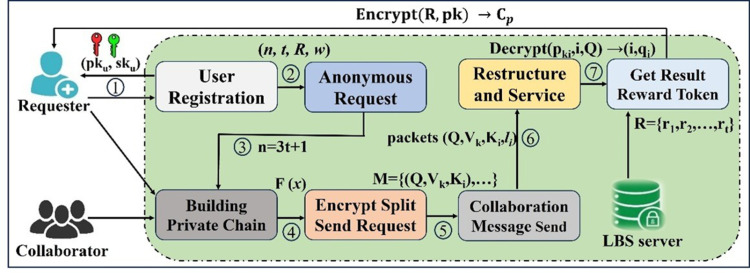
Workflow of anonymous collaboration process.

In the proposed method, the Token value serves as the criterion for prioritizing users’ service requests. Only *t* out of *n* cooperative users can obtain a specific number of Token values, thus encouraging cooperative users to participate more actively and submit collaboration information to the server in a timely manner. This mechanism enhances both the timeliness and success rate of privacy protection. The anonymous collaboration process is as follows: User registration, Anonymous request, Building private chain, Encrypt split and send request, Send collaboration messages, Restructure and service, Get result and Token reward. The workflow is shown in Fig 3.

① User registration. All users register with the CA to obtain a public-private key pair. The private key is securely stored by the user, while the public key serves as identity verification to establish the public license chain.
② Anonymous request. The requesting user publishes collaboration requests in the public license chain, including Token-incentive parameters. Qualified collaborative users who meet the criteria and agree to participate jointly construct temporary private chains with the requester.
③ Building private chain. When users in the public license chain satisfy anonymity requirements and consent to collaborate, they initiate the creation of temporary private chains, thereby becoming designated collaborative users.
④ Encrypt split and send request. The requesting user encrypts the query content and splits the decryption key, sending the encapsulated data packets to collaborative users on the temporary private chain, while transmitting both the actual location and one data packet to the LBS server.
⑤ Send collaboration messages. Collaborative users in the temporary private chain transmit their actual locations and one data packet to the LBS server.
⑥ Restructure and service. Upon receiving at least *t* data packets, the LBS server reconstructs the decryption key, decrypts and verifies the ciphertext information, and retrieves the query request. Subsequently, it performs relevant services and generates corresponding query results.
⑦ Get result and Token reward. The LBS server encrypts the query result with the recovered key and sends it to the temporary private chain. The requesting user obtains the encrypted result from temporary private chain users, then decrypts it to acquire the final query result. The smart contract executes the Token-value assignment function, allocating corresponding Token rewards to the first *t* collaborative users who successfully submitted collaboration information.

## 4. Research contents and method

### 4.1. Basic implementation method

To prevent leakage of location and query privacy while addressing security threats to sensitive personal information (including malicious collusion attacks), this paper proposes a blockchain-based distributed location privacy protection method integrating a secret sharing algorithm and a threshold encryption scheme. The approach safeguards users’ location information by collaborating with *n*-1 cooperative users to generalize real locations, preventing the LBS server from identifying precise positional data. Simultaneously, query contents are encrypted to prevent unauthorized access by other users. A Token-based incentive mechanism introduces coordination rules, encouraging broader user participation in anonymous collaboration while ensuring task completion within specified timeframes.

#### 4.1.1. User registration.

All users are registered with CA (including requesting users and cooperating users), each registered user is assigned a pair of public and private keys, and their identity is stored in the user pool. Public keys are used instead of real identities to complete anonymous requests and collaborations in location-based services, and the public license chain is composed of all registered legitimate users.

① **User service registration**. All users use personal information to register with CA. A random number *r* is selected as a temporary encryption key, and *r* and user identity ID_u_ are encrypted with CA’s public key and sent to CA. After receiving the registration request from the user, the CA decrypts the request with its own private key to obtain the user’s request information, and generates a key pair (pk_u_, sk_u_) for the user. CA uses user temporary key *r* to encrypt key pair (pk_u_, sk_u_) and returns them to user. Finally, the user decrypts the ciphertext with *r* to obtain the key pair (pk_u_, sk_u_), where pk_u_ is the user’s public key and sk_u_ is the user’s private key. The process is as follows:

***Step1***: Input the temporary key *r*, the user identity ID_u_ and the CA’s public key pk_CA_ to generate a request ciphertext:


$$\text{E}({\text{ID}}_{\text{u}}||\text{r})=\text{Encrypt}({\text{ID}}_{\text{u}},\text{r},{\text{pk}}_{\text{CA}})$$ (4)

***Step2***: Input the user’s request ciphertext $E({ID}_{u}||r)$ and the CA’s private key sk_CA_ to obtain the user’s identity ID_u_ and temporary key *r*:


$$\left({\text{ID}}_{\text{u}},\text{r}\right)=\text{Decrypt}(\text{E}({\text{ID}}_{\text{u}}||\text{r}),{\text{sk}}_{\text{CA}})$$ (5)

***Step3***: Input security parameter *λ*, CA’s private key sk_CA_ and user ID_u_ to generate user’s public and private key pair:


$$\left({pk}_{u},{\text{sk}}_{u}\right)=\text{KeyGen}({\lambda ,\text{sk}}_{\text{CA}},{\text{ID}}_{\text{u}})$$ (6)

***Step4***: Input the temporary key *r* and the user’s public and private key pair $\left({pk}_{u},{sk}_{u}\right)$ to generate the user’s key pair ciphertext:


$$\text{E}\left({pk}_{u},{\text{sk}}_{u}\right)=\text{Encrypt}({pk}_{u},{\text{sk}}_{u},r)$$ (7)

***Step5***: Input the temporary key *r* and the ciphertext of the user key pair $E({pk}_{u},{sk}_{u})$ to obtain the public and private key pair of the user:


$$\left({pk}_{u},{\text{sk}}_{u}\right)=\text{Decrypt}(\text{E}({pk}_{u},{\text{sk}}_{u}),r)$$ (8)

② **User authentication**. When an anonymous request is made, the requesting user needs to authenticate to the CA. The user uses his private key sk_u_ to digitally sign the public key pk_u_ representing his identity to obtain ${Sig}_{{SK}_{u}}({pk}_{u})$. Then, he encrypts his own public key pk_u_ and IDu’s digital signature ${Sig}_{{sk}_{u}}({pk}_{u})$ with CA’s public key PK_CA_ to generate an authentication request message and sends it to CA. CA uses its own private key SK_CA_ to decrypt the user request message and verify it. If the verification is successful, an authentication success message is returned.

***Step1***: Input the user’s digital signature ${Sig}_{{sk}_{u}}({pk}_{u})$, public key pk_u_ and CA’s public key PK_CA_ to generate the user’s authentication request ciphertext:


$$\text{E}\left({Sig}_{{sk}_{u}}\left({\text{pk}}_{u}\right),{\text{pk}}_{u}\right)=\text{Encrypt}({Sig}_{{sk}_{u}}({\text{pk}}_{u}),{\text{pk}}_{u},{\text{pk}}_{\text{CA}})$$ (9)

***Step2***: Input the CA’s private key sk_CA_ and the user’s authentication request ciphertext $E({Sig}_{{sk}_{u}}({pk}_{u}),{pk}_{u}$) to obtain the user’s digital signature:


$${Sig}_{{sk}_{u}}\left({pk}_{u}\right)=\text{Decrypt}(\text{E}({Sig}_{{sk}_{u}}({pk}_{u}),{pk}_{u}),{\text{sk}}_{\text{CA}})$$ (10)

#### 4.1.2. Users make anonymous requests.

After the identity of the request user is successfully verified, he can make an anonymous request *q*, and the smart contract will detect his Token value w_u_ and add it to the Token list of the current requesting user. If the request user is in the top *t*, he is allowed to publish collaboration request in the public license chain, the request content comprises the number of cooperative users *n*, the threshold parameter *t*, an anonymous area threshold *R* and a reward Token value *w* and so on. Otherwise, the user needs to wait until his Token value is in the top *t*. To improve the robustness of the method, n = 3t + 1 is set according to Byzantine fault-tolerant mechanism to ensure that enough cooperative users can send cooperative information normally, timely and accurately.

#### 4.1.3. Building temporary private chains based on collaboration request.

**Fig 4:**
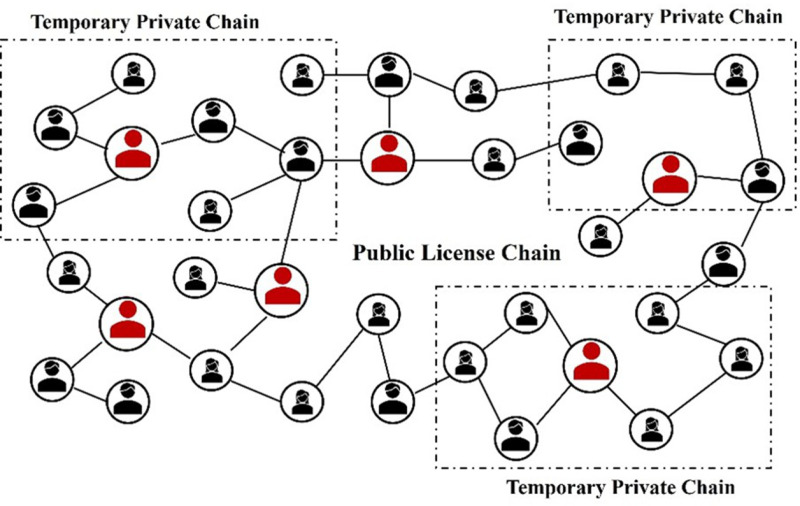
Creating a temporary private chain.

When the users in the public license chain satisfy the anonymity condition and are willing to participate in collaboration, they put forward the request to construct the private chain and become collaborative users. The smart contract executes a predetermined construction protocol to establish a temporary private chain which contains requesting user and cooperating users until the number of users on the temporary private chain is not less than *n*. Collaborative users who meet privacy requirements provide their location information, and the blockchain will create new blocks to record the users’ actions. The temporary private chain created is shown in Fig 4, where all users form a public license chain, and the users in the dashed box are temporary private chains established to satisfy anonymous collaboration requests.

#### 4.1.4. Request user split collaboration request.

After the temporary private chain is created, the requesting user encrypts the request information *q* with the private key sk, and divides the public key pk into n fragments pk_i_ by using the Shamir(t, n) secret sharing method, and then encapsulates them into data packets and sends them to the private chain. The real query information of the request user is defined as q’={lu, c, T}, where *lu* represents the real location, *c* represents the query content, and T represents the timestamp. In order to protect the user’s location privacy, the real location *lu* is hidden, the query information q={c,T} is regenerated, and the private key sk is used to encrypt *q* to generate the ciphertext Q. Then, Shamir(t, n) secret sharing method is adopted to divide the user’s public key pk into *n* public key fragments pk_i_ respectively, and distribute them with the query ciphertext and verification key to n-1 cooperative users on the temporary private chain. The specific process is as follows:

***Step1***: Key generation. Input user’s ID_u_, security parameter *λ*, secret sharing parameters *n* and *t* to generate temporary key pair for the service:


$$\left(p_{k},v_{k},s_{k}\right)=KeyGen(\lambda ,{ID}_{u},n,t)$$ (11)

Where p_k_ is the public key, s_k_ is the private key, and v_k_ is the verification key.

***Step2***: Query content encryption. The request user uses the private key s_k_ to encrypt the query information q={c,T} and generates the request ciphertext Q.


$$Q=Encrypt(s_{k},q)$$ (12)

***Step3***: Split the key. The Shamir (t, n) secret sharing method is used to divide the user’s public key p_k_ into *n* fragments. The specific process is as follows:

① Let a_0_ = k, any t-1 elements a_i_(i = 1,2,…,t-1) are chosen to form a polynomial of order t-1 in GF(p), i.e., ncrypt

$$F(x)=\sum_{i}^{t-1}a_{0}+{ma}_{i}x^{i}modp$$ (13)

Where *p* is a large prime and P > S, secret S = F(0)=a_0_.

② Let x_j_ = k_i_, *n* subkeys can be generated:

$$F\left(k_{j}\right)=F\left(x_{j}\right)=\sum_{i}^{t-1}a_{0}+a_{j}x_{j}^{i}modp$$ (14)

Where j denotes the segment number of the segmentation, j = 1, 2,…,n.

Finally, it can obtain *n* partitioned subkeys (k_i_,F(k_i_)), i.e., {(k_1_,F(k_1_)), (k_2_,F(k_2_)),…, (k_n_,F(k_n_))}. K_i_=(k_i_,F(k_i_)), then *n* subkeys can be represented as {K_1_,K_2_,…,K_n_} respectively.

③ Encapsulated into request packets. The query ciphertext Q, verification key V_k_ and subkey K_i_ are encapsulated into *n* request packets, namely:

$$\text{M}=\{\left(Q,V_{k},K_{1}\right),\left(Q,V_{k},K_{2}\right),\ldots ,\{\left(Q,V_{k},K_{n}\right\}$$ (15)

#### 4.1.5. Send collaboration messages.

The *n*-1 collaborative users are numbered from 1 to n-1 and randomly set to C_1_,C_2_,…,C_n-1_ on the private chain. A hash function H(•) is constructed with each request packet as a variable and modulo it to obtain a cooperative user mapped to number *j*. Then the random mapping mechanism is used to send packets {(Q,V_k_,K_1_),(Q,V_k_,K_2_),…(Q,V_k_,K_n-1_)} to n-1 cooperative users respectively.


$$C_{j}=\text{H}(Q+V_{k}+K_{i}\backslash mod(n-1)$$ (16)

Where 1 ≤ *i* ≤ n, 1 ≤ *j* ≤ n-1.

When different packets are sent to the same cooperating user, conflicts will occur. So it resolves conflicts by setting hash functions:


$$\begin{array}{c} C_{j}=(\text{H}\left(Q+V_{k}+K_{i}\right)+v)mod(n-1) \end{array}$$ (17)

Where v = 1,2,…,n-1 and v = v + 1. At the initial moment, v = 1, and if there is still a conflict among the obtained cooperative user numbers, the *v* value is incremented until the conflict is resolved.

After receiving the data packet (Q,V_k_,K_i_), the cooperative user encapsulates his real position *l*_*i*_ and the received data packet (Q,V_k_,K_i_) into a data packet (Q,V_k_,K_i_,*l*_*i*_) and sends it to the LBS server. In order to ensure that cooperative users can send cooperation information in time, an incentive competition mechanism is introduced, and the first *t* users who send will receive *w* Token value incentive points. The requesting user also combines a data fragment (Q,V_k_,K_i_) and the real location into a data packet (Q,V_k_,K_i_,*l*_*u*_) and sends it to the LBS server. At this time, the LBS server will receive *n* packets containing the same query content ciphertext and different location information.

#### 4.1.6. LBS server obtains query content.

Finally, the LBS server receives *n* packets (Q,V_k_,K_i_,*l*_*i*_) which are sent by the requesting user and the cooperating user. When the number of received packets is not less than *t*, it starts the partial decryption algorithm and the share verification algorithm of threshold encryption to reconstruct the decryption key, decrypt and verify the ciphertext, and obtain a set of query request information.

***Step1***: Recover decryption key p_k_. The LBS server aggregates *t* key fragments {K_1_,K_2_,…,K_t_} in time T, i.e., {(k_1_,F(k_1_)), (k_2_,F(k_2_)),…, (k_t_,F(k_t_))}, it constructs a polynomial of degree *t-1*, and it determines corresponding coefficients a_0_,a_1_,a_2_,…,a_t-1_, and it obtains a polynomial of degree t*-1* with constant coefficients, thereby obtaining the decryption key. The process is as follows:


$$F(xtext={\text{a}}_{0}\text{+}{\text{a}}_{1}\text{x+}{\text{a}}_{2}x^{2}\text{+...+}{\text{a}}_{\text{t-2}}x^{\text{t-2}}\text{+}{\text{a}}_{\text{t-1}}x^{\text{t-1}}$$ (18)

Then the constant coefficient polynomial is obtained as:


$$F(xtext={\text{a}}_{0}\text{+}{\text{a}}_{1}\text{x+}{\text{a}}_{2}x^{2}\text{+...+}{\text{a}}_{\text{t-2}}x^{\text{t-2}}\text{+}{\text{a}}_{\text{t-1}}x^{\text{t-1}}$$ (19)

Let x = 0, F(0)=a_0_, and the constant a_0_ is the decryption key p_k_ requested by the user.

***Step 2***: Partial decryption. In the partial decryption algorithm, the ciphertext Q and the *i*th key fragment *k*_*i*_ are input, and the decryption share (i,q_i_) of the encrypted information is output, specifically:


$$\left(i,q_{i}\right)=Decrypt(P_{k_{i}},i,Q)$$ (20)

***Step3***: Share verification. In the share verification algorithm, a decryption key *p*_*k*_, a verification key *v*_*k*_, a ciphertext Q and a decryption share *q*_*i*_ are input, if the ciphertext is a valid share, a “1” is output, otherwise a “0” is output, specifically:


$$\left(0/1)=Verify(p_{k},{\text{v}}_{k},Q,q_{i}\right)$$ (21)

***Step4***: Share consolidation. In the share association algorithm, the decryption key *p*_*k*_, verification key *v*_*k*_, ciphertext *Q* and *t* decryption shares {q_1_,q_2_,…,q_t_} are input, and the complete request plaintext *q* of the requesting user is output, specifically:


$$\left(q)=Combine(p_{k},{\text{v}}_{k},Q,\{q_{1},q_{2},\ldots ,q_{t}\}\right)$$ (22)

#### 4.1.7. Query and feedback query results and token reward.

After the LBS server decrypts the query request, it first verifies the validity of the timestamp *T* in the query request. If the difference between the timestamp *T* and the system synchronization clock is within the allowable threshold, the request is legal and can be processed.

According to the decrypted query content *q* and *t* query positions *l*_*i*_, LBS server performs query service, so as to obtain *t* query results R={r_1_,r_2_,…,r_t_}. After that, LBS server encrypts the query results with the reconstructed user key *p*_*k*_ to obtain ciphertext *C*_*p*_, and then it sends *C*_*p*_ to the temporary private chain.


$$C_{p}=Encrypt(R,p_{k})$$ (23)

The request user can obtain the query result *C*_*p*_ from the LBS on the private chain, and he can decrypt the query result *C*_*p*_ with his private key s_k_, and he can obtain the final query result according to his location information. Other collaborative users cannot decrypt the query result *C*_*p*_ because they do not have the private key, so they cannot obtain the specific query result.


$$r_{u}=Decrypt(C_{p},l_{u},s_{k})$$ (24)

Finally, the smart contract performs Token allocation function and rewards the first *t* users who successfully send collaboration requests with corresponding Token values. The other *n-t* users get nothing because they don’t respond in time. At the same time, the user is requested to consume *w*_*t*_ Token values. Then the temporary private chain is disbanded and all users return to the public license chain again.

### 4.2. Token incentive system and consensus mechanism

#### 4.2.1. Token incentive system.

A Token is a certificate of equity in digital form that represents an exercisable right within specific scenarios or timeframes. In the paper, a Token incentive system is proposed which uses Token value as the basis for determining whether anonymous collaboration requests can be prioritized, encouraging active user participation.

When a user makes a query request, the higher their Token value, the higher priority their request receives. Upon completion of anonymous collaboration, the requesting user consumes a specified amount of Tokens, while successfully participating collaborators receive allocated Tokens. When initiating a query request, users offering higher Token allocations motivate greater collaboration willingness. However, only collaborators who successfully complete the process obtain tokens, thereby incentivizing timely processing and transmission of collaborative information.

In the proposed method, it is an important reason that collaborative users can get a certain amount of Token value reward, which makes collaborative users more willing to participate in anonymous collaboration and can send collaborative information in time. For any user in the public license chain, the more times he assists other users to successfully complete anonymous collaboration, the more Token reward he receives. When a collaboration user becomes a request user, the number of his Token values determines whether his anonymous collaboration request can be given priority response. At the same time, the larger the Token value set in the collaboration request, the more users willing to participate in collaboration, which would make the anonymity success probability is greater and the collaboration efficiency is higher.

At the initial stage of privacy protection, Token values are published in the public license chain, and each eligible collaborative user can decide whether to participate in collaboration. Then, the request user submits a Token value to the temporary private chain, and the collaborative user sends collaboration information to the LBS server. Finally, after anonymous collaboration is completed, smart contract is activated to reward the collaborative users with Token values. By introducing the incentive competition mechanism, the request user can get the anonymous service in time, the cooperative users can get the Token value, and both parties get the desired result. Therefore, the proposed method can solve the problems that collaboration users are unwilling to collaborate and collaboration information is not sent quickly.

#### 4.2.2. Admission mechanism.

In the paper, a method is proposed to establish an access control mechanism for publicly-permissioned user chains based on blockchain authentication. Only registered users are permitted to join, thereby strengthening information security.

When the user’s request is responded to, a temporary private chain is created. At each request for service, the temporary public key, private key and verification key pair are regenerated by using the user’s private key and privacy parameters to ensure the confidentiality of each request. After the service ends, all temporary keys are cancelled.

#### 4.2.3. Consensus mechanism.

The proposed method establishes a proof-of-stake-based consensus mechanism for private chains, where rights are determined by users’ ownership of tokens. Users gain request privileges by demonstrating Token ownership, with priority responses, master node/leader designation, and transaction accounting authority being proportionally allocated based on Token holdings. Within the public permissioned chain, users receive Token rewards proportional to their frequency of successfully assisting others in achieving anonymization. When initiating anonymous collaboration requests, users may allocate higher Token values to incentivize participation, thereby increasing successful anonymization probabilities. When a requester initiates a request, eligible users add the transaction to their public-chain transaction pools. Responses are triggered if the transaction’s Token value exceeds individual thresholds. A temporary private chain is formed with the requester as master node/leader when responders reach *n*, comprising the requester and *n* − 1 collaborators. Requests failing to meet response thresholds within specified timeframes expire.

When users set (*t*, *n*) secret sharing parameters, *n* ≥ *3t* + *1* is set according to Byzantine Fault Tolerance Mechanism to improve anonymity success rate, so as to ensure that enough cooperative users can send cooperative information normally, timely and accurately. When the request service is successfully completed, the request user consumes a certain Token value, and the collaborative users obtain a certain Token value. The temporary private chain is dissolved, and all users return to the public license chain.

### 4.3. Smart contract approach

The proposed method establishes a proof-of-stake-based consensus mechanism for private chains, where rights are determined by users’ ownership of Tokens. Users obtain request privileges by demonstrating Token ownership, with request priority and master node/leader status being assigned proportionally to token holdings. The accounting authority for transactions is consequently granted to users with higher token balances. Within the public permissioned chain, users earn Token rewards based on their frequency of successfully assisting others with anonymization. When initiating anonymous collaboration requests, users may increase their Token allocation to incentivize participation, thereby enhancing successful anonymization probability. Eligible users add transactions to their public-chain pools when receiving requests. Responses occur when the transaction’s Token value exceeds individual thresholds. A temporary private chain is formed with the requester as master node/leader when responders reach n, comprising the requester and *n*-1 collaborators. Requests failing to meet response thresholds within specified timeframes are marked as expired.

To hide the user’s real location and protect the user’s query content, the request user must establish a temporary private chain with the cooperating user. And request user must first send the collaboration request, anonymity condition and Token reward value in the public license chain. The request user selects the collaborative users based on the collaborative anonymous condition, it must ensure that the selected location could generalize his real location. Request user sends *n-1* sets of encrypted data packets to the private chain. Each collaborative user must send the ciphertext data packet and their real location to the LBS server in the private chain. When the number of collaborative users is large enough, each collaborative user must send collaboration information to the LBS server in time, otherwise he will not get Token rewards. Because only the first *t* collaboration users who send collaboration information can get Token value reward. Therefore, the proposed method can ensure that anonymous requests of user can be quickly responded and collaborated.

The LBS server encrypts the result set with the public key of the request user and publishes it to the private chain, and all users on the private chain can obtain the encrypted result. Only the request user has the corresponding private key, he can decrypt it and extract the desired result. Other collaborative users on the private chain cannot decrypt the result set without decrypting the private key, and they cannot obtain any information of the request user. In addition, the LBS server receives at least *t* locations for the same query content, and it cannot determine the exact location of the request user.

In the process of request user to establish a private chain with the collaborative user, and in the process of generalizing the real location with the collaborative user, the smart contract mainly performs two functions. On the one hand, after the request user sends the anonymous request in the public permission chain, it establishes a temporary private chain and joins the cooperating users who meet the conditions into the private chain. On the other hand, it examines the result set and rewards the first *t* collaboration users who successfully participate in collaboration. The process can be described in Algorithm 1. 
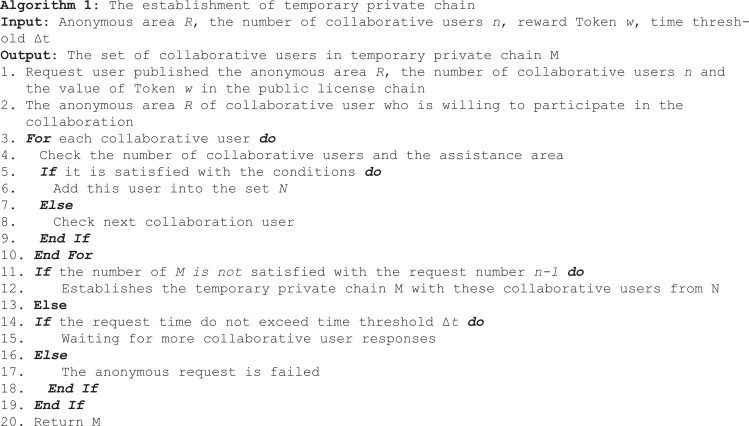


After the temporary private chain is established, the information of the request user and the cooperate user is recorded, and then the request user sends the encrypted information fragment to the cooperate user in the private chain. All collaborative users send encrypted pieces of information and their real location to the LBS server. After the LBS server completes information verification, reconstruction, query and encryption, it feeds back the result set to the temporary private chain. The process can be described in Algorithm 2. 
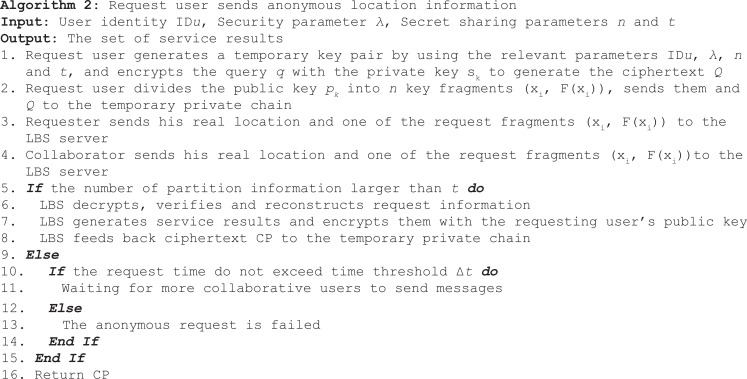


Smart contracts can help request user extract results from collections and reward collaborative users. Algorithm 3 describes the process of extracting results and rewarding collaborative users. 
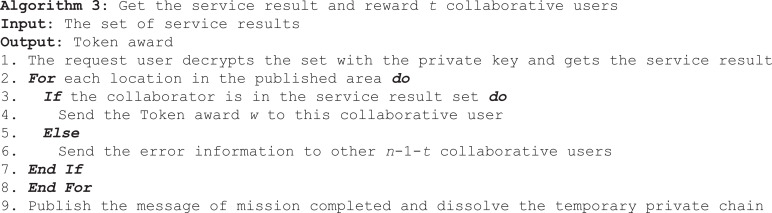


In algorithm 3, the request user gets the service result, and the cooperate users get the reward Token value, which is recorded in the blockchain and cannot be changed. In the process of sending the request and obtaining the result, the position of request user is generalized, the query content is encrypted, the decryption key is divided, and the query result is encrypted, it is difficult for collaborative users to obtain the true information of request user. In addition, the Token value is used as the basis for the user’s anonymous request to be preferentially responded, and only part of the cooperative users can obtain the reward Token value. The competition mechanism can inspire collaborative users to actively participate, they can quickly send collaborative information to the LBS server, and it can ensure that the request users get the service results in time.

## 5. Analysis of privacy protection effect

### 5.1. Security analysis

#### 5.1.1. Information confidentiality.

The requester encrypts the query content and divides the decryption key among *n*-1 cooperative users. Since cooperative users cannot reconstruct the decryption key or access the specific query content, this process ensures query confidentiality. The user directly sends their real location to the LBS server, preventing cooperative users from obtaining this information. When the LBS server receives at least *t* pieces of cooperation information, it can reconstruct the decryption key and obtain *t* real locations with accurate query content. However, the server cannot determine which location corresponds to the actual requester nor link the query to the real user. Therefore, the probability of identifying the requester’s real location does not exceed 1/*t*, ensuring location confidentiality. Following the Byzantine fault-tolerant mechanism, parameter *t* is set as *t*=(*n*-1)/3 to prevent collusion attacks by malicious users and maintain privacy confidentiality. The LBS encrypts query results with the requester’s public key, which cooperative users cannot decrypt, thus ensuring end-to-end information security.

#### 5.1.2. Information integrity.

The proposed method employs key partitioning technology to generate verification keys, utilizing key verification and share-union algorithms to validate received ciphertext fragments. This ensures information integrity and unforgeability.

#### 5.1.3. Key security.

The requester’s query content undergoes asymmetric encryption, with the decryption key divided into *n* fragments distributed among *n* participants. Since no single participant can reconstruct the complete key, the decryption key remains secure.

#### 5.1.4. Eavesdropping attack.

An eavesdropping attack occurs when attackers listen to communication channels to intercept sensitive unencrypted information. In our proposed method, both the requester’s query content and LBS responses are encrypted during transmission. Even if attackers acquire the encrypted data through eavesdropping, they cannot decipher the original content.

#### 5.1.5. Replay attack.

Replay attacks occur when malicious nodes spoof LBS server’s trust by redistributing legitimate packets. In our proposed method, each request ciphertext contains a timestamp *T*. Upon receiving requests, the LBS server first verifies whether the difference between its synchronized clock and *T* exceeds the predefined threshold. Requests are only processed when the temporal discrepancy remains within the allowable range. This mechanism effectively resists replay attacks.

### 5.2. Computational complexity analysis

The proposed method involves information encryption/decryption, secret sharing, and digital signature operations. Considering digital signatures as a specialized form of encryption/decryption operations (where decryption is the inverse of encryption), we define O(Sha) as the computational complexity for secret sharing, and O(Enc) as the combined complexity for encryption, decryption, and digital signatures.

In the proposed method, secret sharing requires *n* public values and constructs *t* interpolation functions, while secret recovery involves solving *t* equations. This results in an overall computational complexity of O(*n* + *t*). Therefore, the computational complexity *p*_1_ for secret sharing can be expressed as:


$$p_{1}=\text{O}(Sha)=\text{O}(n+t)=\text{O}(n)$$ (25)

In the proposed method, when the CA receives a user request, it first verifies the signature data ($E({Sig}_{{sk}_{u}}({pk}_{u})||{pk}_{u}$)) using its private key sk_CA_, requiring O(Enc) computational complexity. If verification fails, the anonymous request terminates. Successful verification triggers generation of a temporary key pair for the user. The user encrypts query content with this key pair and broadcasts the encrypted data packet through the temporary private chain. The time complexity *p*_2_ comprises:


$$p_{2}=\text{O}(n-1)+\text{O}(Enc)+\text{O}(Enc)+\text{O}(Enc)=\text{O}(Enc)$$ (26)

Upon receiving a data packet, the collaborative user forwards both their real location and the packet to the LBS server, requiring O(1) computational complexity. When the LBS server accumulates at least *t* data packets, it initiates key reconstruction. This process enables the server to retrieve the authentic query request, generate corresponding results, encrypt these results, and broadcast the ciphertext through the temporary private chain. The requester then decrypts the chain’s ciphertext to obtain the query results. The required computational complexity *p*_3_ is:


$$p_{3}=\text{O}(t)+\text{O}(Enc)+\text{O}(Enc)+\text{O}(Enc)=\text{O}(Enc)$$ (27)

To sum up, the upper bound of computational complexity of the proposed method is:


$${p=p_{1}+p_{2}+p}_{3}=\text{O}(n)+\text{O}(Enc)+\text{O}(Enc)=\text{O}(Enc)$$ (28)

### 5.3. Efficiency analysis

#### 5.3.1. Method for protecting user’s location privacy.

Existing user location privacy protection methods [19–23] typically aggregate the requester’s real location with *k*-1 other users’ locations to construct anonymous zones. These centralized server-based approaches, where the server manages anonymous zone construction and request processing, create system bottlenecks that undermine privacy protection effectiveness. Although methods [29–34] propose distributed alternatives requiring requesters to collaborate peer-to-peer with *k*-1 users, thereby reducing server load while protecting location privacy, collaborative users may be unwilling to share their real locations. This paper introduces a Token incentive system that prioritizes anonymous collaboration requests based on Token value, encouraging active participation. In this scheme, users directly send their real locations to the LBS server, preventing collaborators from accessing this information. The requester protects location privacy through collaborative generalization of their real location. When the LBS server gathers at least *t* cooperation records, it cannot identify which location corresponds to the actual requester, resulting in a recognition probability not exceeding 1/*t*. This mechanism ensures real location confidentiality through parameter *t*=(*n*-1)/3, derived from Byzantine fault tolerance principles.

#### 5.3.2. Method for protecting user’s query content privacy.

While most existing privacy protection methods [28–32] (e.g., k-anonymity and differential privacy) can safeguard users’ location privacy, they fail to protect query content privacy. In anonymous collaboration systems, participants may access others’ query content even when location information is generalized, posing content leakage risks. Conversely, methods in References [24–26] employ cryptographic techniques to dissociate query content from users, thereby protecting query privacy at the expense of exposing location information. This dichotomy stems from conventional approaches that isolate location and query information protection. In this paper, the proposed method achieves dual-layer privacy protection through two mechanisms, location generalization via distributed collaboration to preserve location privacy, and query content encryption using secret-sharing and threshold cryptography to partition and secure query content.

In summary, the proposed method not only protects the user’s location privacy but also their query privacy by solving privacy leakage problems caused by the unwillingness of collaborative users, unreliable location services, and delayed delivery of collaborative information. A comparison between the proposed method and existing location privacy-preserving methods is presented in Table 1.

**Table 1:** Comparison of method effectiveness.

Method	location privacy	query privacy	Collaborative user management and incentives
Proposed method	√	√	√
[19–23]	√	×	×
[24–26]	×	√	×
[28–32]	√	×	×
[33,34]	×	√	×
[27,34,37]	√	×	√

#### 5.3.3. Communication overhead.

Communication overhead is formally defined as *the temporal and computational resource consumption during distributed data exchange processes*, encompassing factors such as data transmission latency, network propagation delay, and packet payload size. As an irreducible constraint in distributed systems, this metric is typically analyzed through asymptotic time complexity frameworks. Specifically, when a requester initiates multi-party communication, the collective time complexity increases monotonically with communication overhead magnitude, establishing it as a pivotal performance metric for distributed algorithms.

In the proposed method, the requesting user transmits partitioned data packets to collaborative users via a transient private chain. These packets are strictly limited to the user’s request content and threshold-fragmented cryptographic shares, both engineered for minimal byte-level footprints. This lightweight payload design ensures negligible transmission latency and network propagation delay, resulting in statistically insignificant communication overhead. Consequently, the primary system overhead originates from cryptographic computations rather than data transmission. Through asymptotic complexity analysis, the peak computational overhead is attributed to four core operations: query encryption/decryption, digital signature generation, signature verification, and threshold secret reconstruction. Therefore, the communication overhead is asymptotically bounded by O(*Enc*), where *Enc* denotes the complexity of the underlying encryption primitive.

## 6. Experimental evaluation

In this section, the proposed method’s performance is evaluated through systematic configuration of privacy-preserving parameters to assess its effectiveness. Simultaneously, comparative analysis with the other three methods was conducted to validate the feasibility and effectiveness of the approach.

### 6.1. Experiment setting

The experiment employs Elliptic Curve Cryptography (ECC) for encrypting requesters’ query content. As the most suitable encryption and signature algorithm for mobile devices, ECC offers lower computational overhead and higher security levels compared to other public-key cryptography schemes. For secret distribution, the Shamir secret-sharing mechanism utilizes t-order polynomial construction, while threshold cryptography enables secure secret sharing among multiple participants. Accordingly, the Shamir secret-sharing algorithm was implemented for decryption key partitioning, with parameters set as n = 3t + 1 where *n* (number of cooperative users) ranges from 7 to 25 and *t* (threshold) ranges from 3 to 8. A distributed permissioned blockchain was built using Ethereum 1.5 – an open-source, modular, smart-contract-enabled blockchain platform. The network comprised 25 nodes, *p*_0_ is as the requester node and (*p*_1_,*p*_2_,…,*p*_24_) are as cooperative nodes. When *p*_0_ initiates an anonymous request, a minimum of *n-1* cooperative users establish a temporary private chain. The encrypted request information and split decryption keys are then distributed to collaborating users.

In the experiment, the number of anonymous cooperative users *n* ranges from 7 to 25 (7 ≤ n ≤ 25), while the encryption threshold *t* varies between 3 and 8 (3 ≤ t ≤ 8). For each parameter set, the execution was repeated 100 times with the average value recorded as the final result. The experimental environment consisted of an Intel(R) Core(TM) i7 CPU@5.4 GHz, 32GB RAM, and a Windows 11 64-bit operating system. The algorithm was implemented in Java using the JPBC 2.0 Cryptographic Library, which provides extensive predefined cryptographic computations and is widely adopted in Java programming environments. The experimental data originated from the Geolife dataset [41–43], containing 24,876,978 location points and 18,670 trajectories collected from 182 users engaged in various outdoor activities. Each location point includes latitude, longitude, timestamp, and other attributes, collectively covering 1,292,951 km. While most data was collected in Beijing, a small portion originates from Europe and the United States.

### 6.2. Experiment and result analysis

This section evaluates the proposed method by configuring diverse privacy preservation parameters, including: the number of collaborative users *n*, secret sharing threshold *t*, Token incentive values, and tolerance time threshold Δ*t*. The experimental framework assesses performance through execution time and anonymity success rate. Furthermore, comparative analysis with three baseline methods validates the method’s feasibility and effectiveness. Scheme1 implements *k*-anonymity by distributing real-time location data to *k* anonymizers, with randomized selection of one anonymizer to forward obfuscated location information to the LBS server [44]. Scheme 2 constructs *k*-anonymous sets through behavioral similarity clustering based on user activity patterns and Points of Interest (POIs), thereby obscuring actual location data within homogeneous user groups [45]. Scheme 3 employs homomorphic encryption for location data before transmission to proxy nodes, ensuring proxy nodes execute anonymization without accessing plaintext private information [46].

#### 6.2.1. Comparison of execution time and anonymity success rate for different numbers of collaborative users.

**Fig 5:**
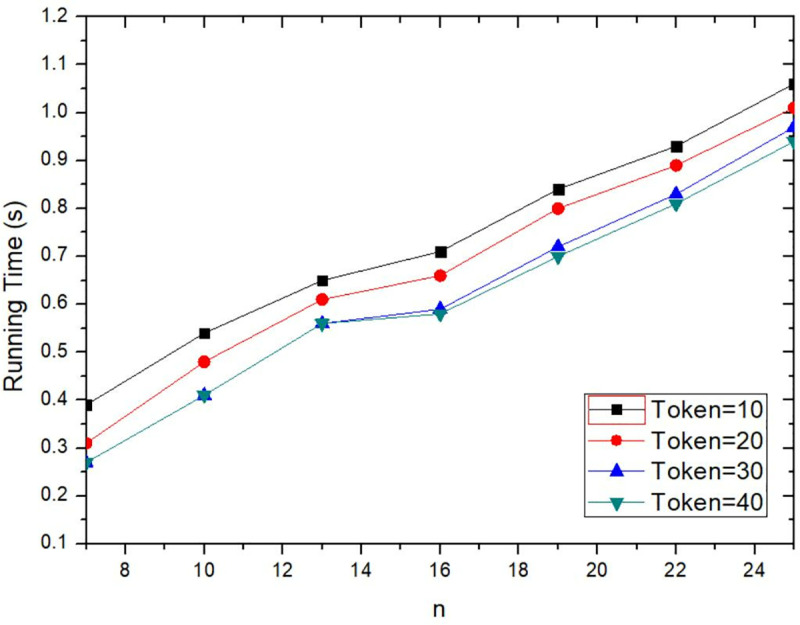
Comparison of execution time for different collaborative users.

The execution time of the proposed method is rose with the increasing value of *n*, as shown in Fig 5. The execution time starts from publishing the user’s request to obtaining the service result. The specific process includes that user puts forward a cooperation request, encrypts the data, divides key and sends the data, the cooperation users send cooperation information, the LBS server reconstructs key and decrypts data, verifies the request, performs query service and returns service results, and the request user to obtain query results. As *n* is increased, the more users are required to participate in collaboration, so that the execution time for selecting collaborative users is increased. As can be seen from the Fig 5, when the Token value is constant, the larger *n* value, the longer execution time. When *n* is constant, the larger Token value, the shorter execution time. When the Token incentive value is increased, the more users are willing to participate in anonymous collaboration, the user’s choice time is shorter. When the number of collaborative users *n* is set within a reasonable range, the execution time will be increased, but it will not change too much. Because collaborative users decide whether to participate in collaboration according to Token value and anonymity requirement, it only compares whether the relevant threshold meets the condition, and the computational complexity is not high.

**Fig 6:**
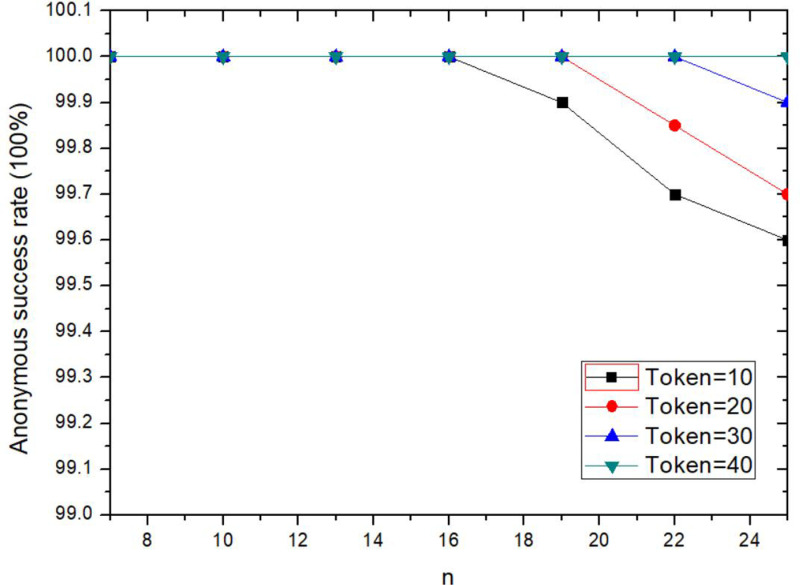
Comparison of anonymity success rates for different collaborative users.

The anonymity success rate of the proposed method is decreased with increasing value of *n*, but it does not change significantly within a certain range (n < 18), as shown in Fig 6. Anonymous success means that there are no less than *n*-*1* collaborative users participating in the anonymity, so that the user’s real information cannot be identified. When *n* is increased, it means that the number of collaborative users participating in anonymity is increased, and the privacy protection effect is further improved. At the same time, more collaborative users need to respond to anonymous request and participate in anonymous collaboration. The increase of *n* value makes the anonymity requirement further enhanced, which makes the difficulty of anonymity success increased and affects the anonymity success rate. As can be seen from the Fig 6, when the incentive Token = 40, the anonymity success rate is 100%. When Token≤20 and n ≥ 18, the success rate of anonymity is decreased slightly. In the experiment, the response threshold of the collaborative user is randomly set, when the Token value and anonymity condition cannot both meet the requirement of the collaborative user, anonymity would be failed. Therefore, when *n* is increased, the anonymity success rate is affected, it shows that setting incentive parameter Token and constructing incentive mechanism play an important role in privacy protection.

#### 6.2.2. Comparison of execution time and anonymity success rate with different threshold.

**Fig 7:**
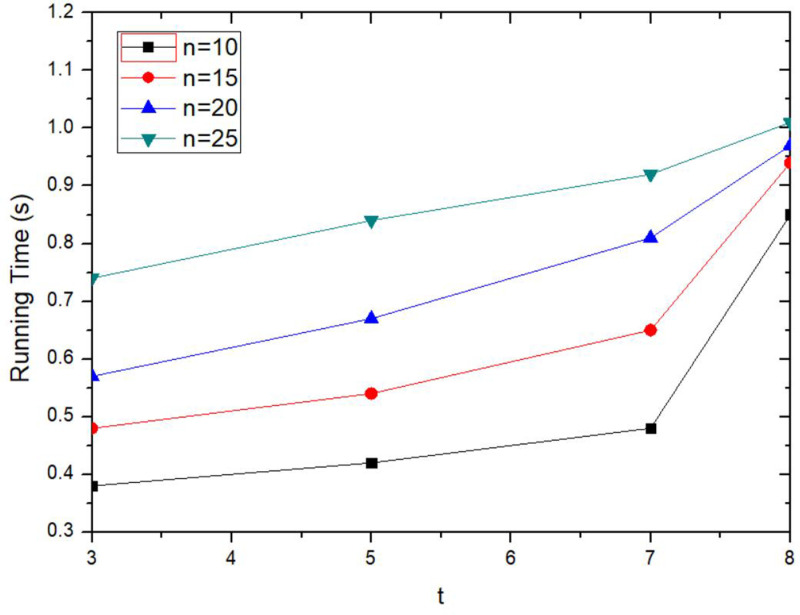
Comparison of execution time for different thresholds.

The execution time of the proposed method is boosted with the increasing threshold *t*, as shown in Fig 7. The threshold *t* represents the minimum share of key fragments required for key recovery. The larger value of *t*, the more collaborative users are required to send quickly collaboration information. The longer LBS server waits for collaboration users to send collaboration information, the longer execution time. As can be seen from the Fig 7, when *t* is increased, the execution time is also increased. When *t* is constant, the execution time is up with the number of anonymous collaboration users *n* increases, it shows that the collaborative user response and selection phase takes a higher proportion of execution time than the send request phase. When *n* is larger, the execution time varies more slowly with the threshold *t*. It further illustrates the effectiveness of Token incentive mechanism. When collaborative users are willing to participate in anonymous collaboration, they will send collaboration information in time to obtain Token rewards.

**Fig 8:**
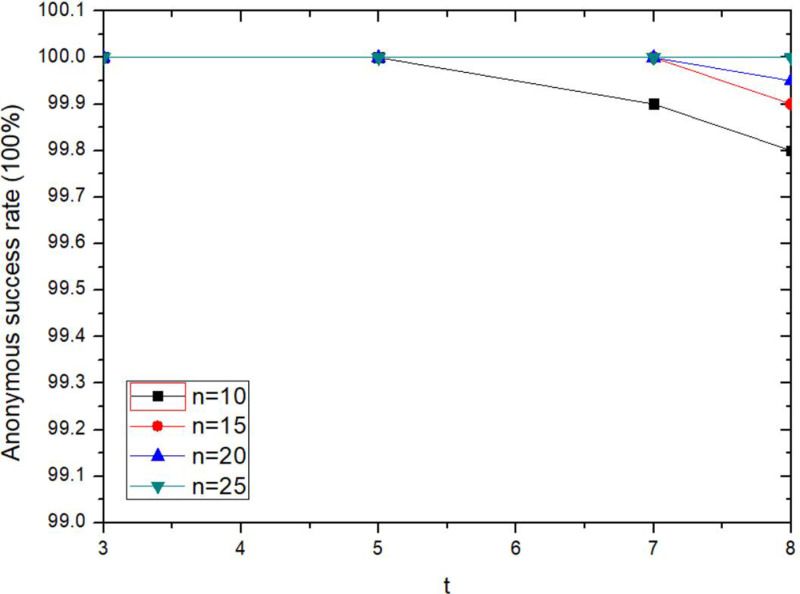
Comparison of anonymity success rate with different thresholds.

The anonymity success rate of the proposed method does not change significantly with the increasing threshold *t*, as shown in Fig 8. The larger *t* is, the more key fragment shares are needed to recover the decryption key, which will affect the anonymity success rate. When the Token value meets the incentive threshold of collaborative users, they will participate in the construction of temporary private chain. If and only if the number of collaboration users is not less than *n*-*1*, the collaboration anonymization would be successful. As can be seen from the Fig 8, when *t* is constant, the larger *n*, the higher success rate of anonymity. When n ≥ 20, the anonymity success rate is 100%.

#### 6.2.3. Comparison of execution time and anonymity success rate for different Token.

**Fig 9:**
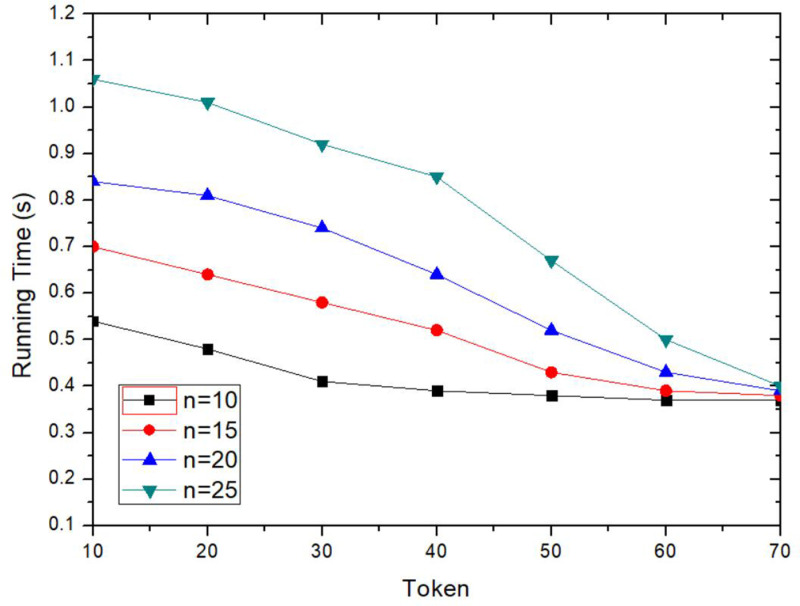
Comparison of execution time for different Token.

The execution time of the proposed method is decreased with the increasing of Token value, as shown in Fig 9. Token value is an important incentive mechanism to encourage collaborative users to participate in anonymous collaboration. The larger Token value of the request user, the more preferentially of his anonymous collaboration request is prioritized. The larger Token reward for request user to publish, the larger reward for collaborating users after successful collaboration. Therefore, collaboration users are more willing to participate in collaboration and can send collaboration information in time to get more Token values, which will reduce execution time. As can be seen from the Fig 9, when Token≥60, and *n* is 10 and 25, the execution time does not change significantly. When Token is greater than the threshold set by most collaboration users, many users are willing to participate in collaboration.

**Fig 10:**
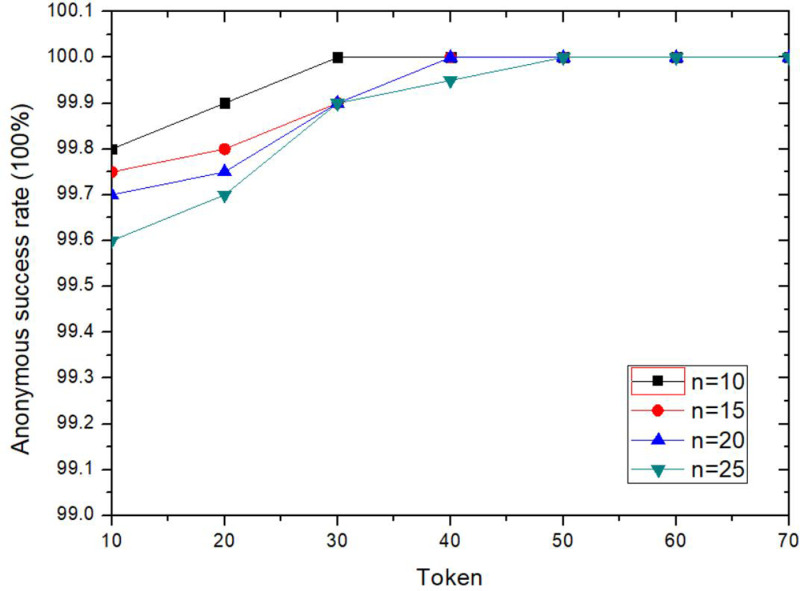
Comparison of anonymity success rates for different Token.

The anonymity success rate of the proposed method is shoot up with the increasing of Token value, as shown in Fig 10. The higher Token value of the request user, the priority will be given to his anonymous collaboration request, and the higher success rate of anonymity. The larger Token value published, the more collaborative users are willing to participate in anonymous collaboration, and the higher success rate of anonymity. As can be seen from the Fig 10, when Token≥50, and *n* is reasonable, more users will be willing to participate in collaboration, and the anonymous success rate is 100%.

#### 6.2.4. Comparison of anonymity success rates for different tolerance time threshold.

**Fig 11:**
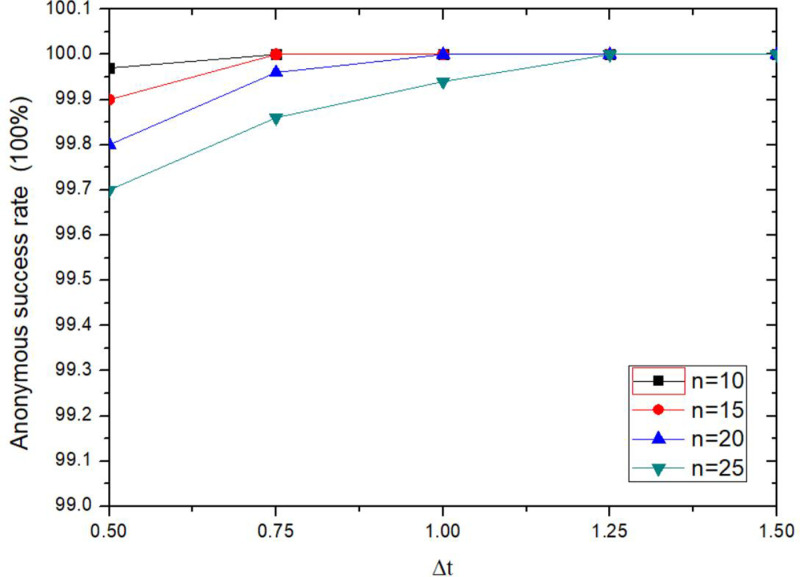
Comparison of anonymity success rates for different tolerance time collaborative user.

The anonymity success rate of the proposed method is increased with the increasing of tolerance time threshold Δ*t*, as shown in Fig 11. It sets a maximum tolerance time threshold Δ*t* for anonymous collaboration, when the execution time is longer than Δt, the anonymous collaboration is not completed, the anonymous collaboration is failed, and the anonymous process will be terminated. The larger tolerance time threshold Δ*t*, the higher success rate of anonymity to some extent, but it will not have too much impact. The willingness of a collaborative user to participate in anonymous collaboration is affected by many factors such as anonymity conditions, Token values and so on. If the Token value does not meet the threshold of collaborative user, he will not participate in anonymous collaboration. Increasing the tolerance time threshold Δ*t* is only one of the factors to improve the success of anonymity. It can be seen from the Fig 11, when Δt ≤ 0.75s, and n ≥ 15, the anonymous success rate is less than 100%. When Δt ≥ 1.25s, for all parameters *n*, the anonymity success rate is 100%.

**Fig 12:**
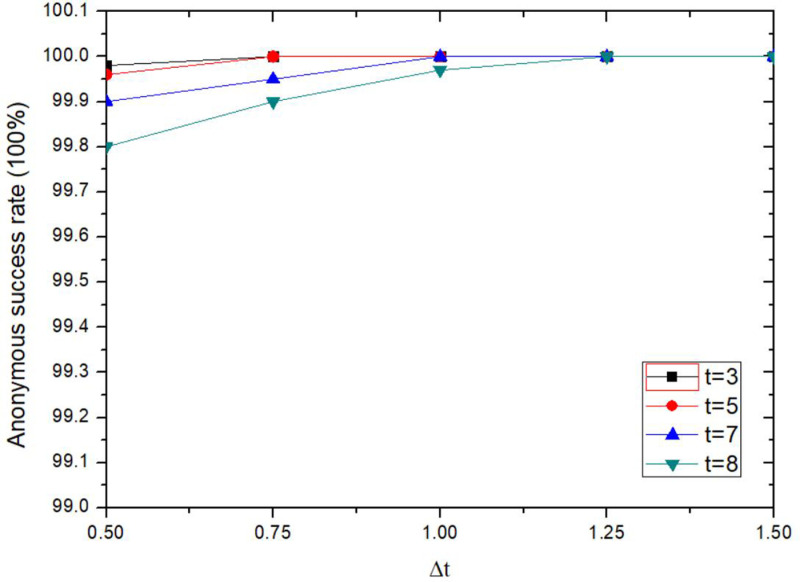
Comparison of anonymity success rates for different tolerance time threshold.

The anonymity success rate of the proposed method is increased with the increasing of tolerance time threshold Δ*t*, it is also affected by Shamir’s secret sharing threshold *t*, as shown in Fig 12. When the tolerance time threshold Δ*t* is constant, the success rate of anonymity will be decreased when the secret sharing threshold *t* is increased. When Δt ≥ 1.25s, for the set secret sharing threshold *t*, the success rate of anonymity is 100%. Because *t* represents the minimum share of key fragments required for key recovery using Shamir secret sharing method, the more shares are needed, the more collaborative users need to send collaboration information quickly. The longer the LBS server waits, the longer the anonymity lasts. If the tolerance time threshold Δ*t* is unreasonable, anonymous collaboration could be failed.

#### 6.2.5. Comparison of execution time and anonymous success rate of different methods.

Traditional privacy protection methods primarily include approaches such as k-anonymity, differential privacy, and data encryption. These methods either employ k-anonymity techniques to construct anonymous regions or sets through generalization, perturbation or suppression to protect users’ private information, or utilize encryption to process user requests and query content, thereby preventing privacy leaks. In this paper, the proposed method is compared with three traditional privacy protection methods (Scheme 1, Scheme 2, and Scheme 3) to validate its feasibility and effectiveness.

**Fig 13:**
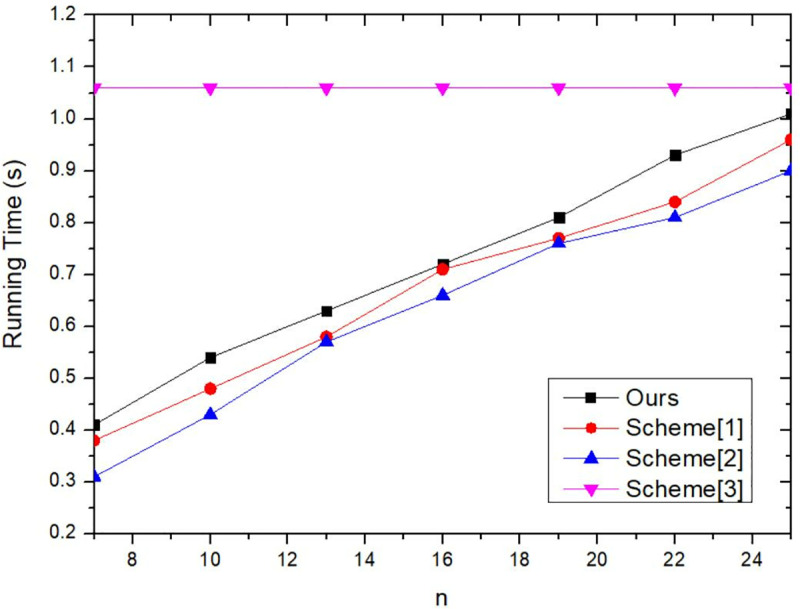
Comparison of execution time of different methods.

The execution time of the proposed method is compared with the other three methods, as shown in Fig 13. Two methods (Scheme 1 and Scheme 2) protect privacy information through collaborative anonymity, and one method (Scheme 3) achieves this through encryption. It can be seen that the execution time of the three collaborative anonymity methods, including the proposed method, increases with the value of *n*, while the execution time of the encryption method remains constant regardless of *n* but is longer than those of the other three methods. When *n* increases, more users need to participate in collaboration, leading to increased time for selecting collaborative users and consequently longer execution time for collaborative anonymity methods. The execution time of the proposed method is slightly longer than those of the other two collaborative anonymity methods. The encryption method protects privacy information through encryption, which is independent of the number of cooperative users, but its execution time is significantly longer.

In the proposed method, the collaborative user decides whether to participate in collaboration based on the Token value and anonymity conditions of the request user. It only verifies whether the relevant thresholds are satisfied, resulting in low computational complexity, therefore, the number of collaborating users has minimal impact on execution time. After selecting the collaborative users, only the user’s request content is encrypted, which involves a small data volume and low computational complexity. Key splitting, key reconstruction and data decryption require limited computation, so the execution time does not increase significantly compared to the other two cooperative anonymity methods. With advances in hardware performance, the encryption and decryption operations of the proposed method are executed by high-performance hardware devices, the execution efficiency can be substantially improved, and the impact on anonymous users will be further reduced.

**Fig 14:**
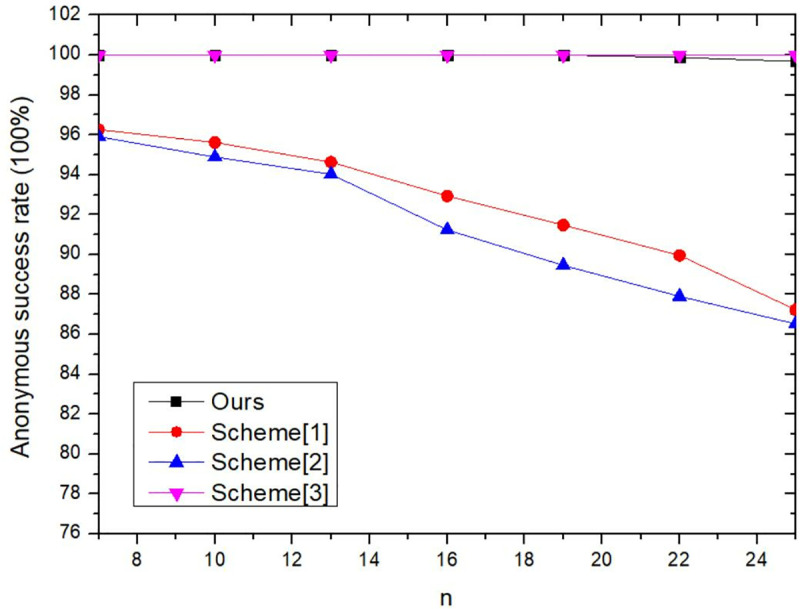
Comparison of anonymization success rates for different methods.

The anonymous success rate of the proposed method is compared with the other three methods, as shown in Fig 14. As shown in the figure, the anonymity success rate of the two comparative collaborative anonymity methods decreases significantly as *n* increases, while that of the proposed method declines slightly with increasing *n*. In contrast, the anonymity success rate of the encryption method remains constant regardless of *n*. For the proposed method and the other two collaborative anonymity methods, an increase in *n* implies that more users must participate in anonymization and more collaborative users are required to respond to requests, thereby increasing the difficulty of selecting qualified collaborators and ultimately reducing the anonymity success rate.

As illustrated in Fig 14, the proposed method demonstrates a higher anonymity success rate compared to the two baseline collaborative anonymity methods. This paper introduces a Token-based incentive mechanism that incentivizes higher rates of user participation in collaborative anonymity and ensures timely delivery of anonymization responses, thereby enhancing overall anonymity success rates. However, the proposed method exhibits a marginally lower success rate than the encryption-based approach. When the requester’s Token value falls below a critical threshold, or when the number of responding collaborators is insufficient due to low Token incentives, the anonymity will be failed. Notably, as *n* increases, both baseline collaborative methods experience significant degradation in anonymity success rates, whereas the proposed method maintains superior performance through its Token-driven incentive framework.

## 7. Conclusions and next work

To solve the privacy leakage problems caused by the unwillingness of collaborative users, unreliable location services, and delayed delivery of collaborative information, a location privacy protection method based on blockchain technology and threshold cryptography mechanism is proposed in this paper, which can realize double protection of users’ location privacy and query privacy. The method constructs a distributed location privacy protection framework based on threshold cryptography, it encrypts query content, divides the decryption key, and generalizes the real location. This approach can solve the problem of incomplete trust in third parties within location-based services. The method constructs a blockchain-based privacy protection mechanism that uses smart contracts to create temporary collaborative private chains to ensure confidentiality during information transmission. It uses Token value as an equity proof to build a user consensus mechanism, where request users with higher token values are prioritized. It implements a competition mechanism to encourage collaborating users to provide collaboration information promptly, solving the privacy leakage problem caused by the unwillingness of anonymous collaborators to cooperate and delayed collaboration information delivery. The method sets privacy protection parameters based on the Byzantine fault-tolerant mechanism to improve the robustness of the privacy protection framework. By introducing a verification key, the method uses a key verification algorithm and a share association algorithm to ensure the integrity and availability of transmitted information, addressing the privacy leakage problem caused by collusion attacks. Finally, the feasibility and effectiveness of the proposed method are verified on real datasets.

However, since the proposed method uses information encryption and key splitting to protect privacy data, its efficiency shows limited improvement. In future work, we will improve the algorithm’s execution efficiency to enable effective application in diverse scenarios.

## Supporting information

S1 File Data. (ZIP)
